# Supplementary material for: Chronotype-specific associations of sleep–wake schedule with behavioral, physiological, and psychological parameters
Source: Sleep Adv. 2026 Aug 18;7(3):zpag087. doi: 10.1093/sleepadvances/zpag087 (PMC13570647; doi:10.1093/sleepadvances/zpag087)
Supplement: Supplementary file 1 [file supplementary_files_zpag087.zip › haga_Supplementary_file_NOT_for_review_final_zpag087.docx]

**Chronotype-specific associations of sleep-wake schedule with behavioral, physiological and psychological parameters**

Toko Haga^1^, Seitaro Iwama^2^, Junichi Ushiba^2^, Junichi Ushiyama^3,4^*

^1^Graduate School of Media and Governance, Keio University, Fujisawa, Kanagawa, Japan

^2^Department of Biosciences and Informatics, Faculty of Science and Technology, Keio University, Yokohama, Kanagawa, Japan

^3^Faculty of Environment and Information Studies, Keio University, Fujisawa, Kanagawa, Japan

^4^Department of Rehabilitation Medicine, Keio University School of Medicine, Tokyo, Japan

***Correspondence**

Junichi Ushiyama

Email: ushiyama@sfc.keio.ac.jp

<https://orcid.org/0000-0001-5947-2553>

**・Signal quality**

To demonstrate signal quality, we added a new figure (Figure S1) illustrating both representative data from an individual participant and group-level averaged results.

The power spectral density exhibited a typical 1/f-like decrease with frequency, and most participants showed a clear peak in the alpha band. These features are consistent with physiologically plausible EEG signals and qualitatively indicate that the recordings were obtained with sufficient signal quality.

**・Electrode placement consistency**

The headphone-type device was adjusted so that the earlier cups were positioned directly above the ears, resulting in automatic placement of electrodes near C3, Cz, and C4. Furthermore, we now cite our previous preprint^1^, which demonstrated that signals obtained with this device show significant correlations with conventional EEG recordings at C3, Cz, and C4 in most participants. The proposed headset maintained electrode locations within 8 mm of the ideal sensorimotor positions, demonstrating high placement consistency compared to other commercially available EEG headsets^2^.

**・Impedance and noise characteristics**

Impedance values were continuously recorded during data acquisition and subsequently analyzed. The impedance data are presented in Figure S2. For EEG systems with high-input-impedance amplifiers, the commonly recommended impedance range is approximately 40–50 kΩ^3^.

A total of 120 EEG data segments (4 sessions × 15 participants × 2 groups) per electrode were obtained for Cz, C3, and C4. After applying the exclusion criterion (impedance > 50 kΩ), 6, 20, and 10 segments were excluded for Cz, C3, and C4, respectively. After exclusion, the obtained impedance values were as follows:

・Cz: 25.97 ± 11.30 kΩ
・C3: 28.42 ± 10.30 kΩ
・C4: 24.85 ± 9.95 kΩ

These values fall within or below the recommended impedance range for high-input-impedance EEG systems, suggesting that sufficient electrode–skin contact was achieved. In addition, the data segments with impedance exceeding 50 kΩ were excluded, and all analyses were repeated based on this criterion^3^. The corresponding data analysis, results and figures have been updated accordingly. Importantly, the exclusion of high-impedance segments did not materially change the overall results or statistical conclusions.

**・Agreement with standard scalp EEG**

In our previous experiment^1^, evaluation of the quality of motor task event-related desynchronization (ERD) during a motor execution task was assessed using both the proposed electrode holder and standard scalp EEG. The alpha-band center frequencies showed a very high correlation (r = 0.97), indicating strong agreement with standard scalp EEG.

**・Test-retest reliability ICC**

We evaluated within-subject consistency of EEG power across repeated measurements using intraclass correlation coefficients (ICC). The analysis was conducted for both alpha and theta bands. ICC values were calculated using publicly available MATLAB code (<https://jp.mathworks.com/matlabcentral/fileexchange/22099-intraclass-correlation-coefficient-icc>).

・Cz: alpha, ICC = 0.500; theta, ICC = 0.420 (p < 0.001)

・C3: alpha, ICC = 0.526; theta, ICC = 0.345 (p < 0.001)

・C4: alpha, ICC = 0.650; theta, ICC = 0.480 (p < 0.001)

ICC values above 0.7 are considered indicative of high reliability. In the present study, alpha-band power showed moderate reliability, whereas theta-band power showed lower consistency. Importantly, EEG recordings were obtained at different time points (e.g., morning and evening). Therefore, the reduced ICC in the theta band likely reflects genuine physiological variability rather than measurement instability. This interpretation is consistent with our main finding that theta power varies as a function of time. These results suggest that the device captures both relatively stable components (alpha) and state-dependent fluctuations (theta).

**・failure rates**

The failure rate was defined as the proportion of EEG data segments excluded due to excessive impedance (>50 kΩ). The failure rate was 5.0% for Cz (6/120), 16.7% for C3 (20/120) and 8.3% for C4 (10/120).

**Detailed signal processing procedures:**

**・Artifact rejection**

Artifact-contaminated segments were identified by visual inspection and excluded from further analysis. The total duration of rejected data was 72 s for Cz, 94 s for C3, and 110 s for C4, corresponding to 0.50%, 0.65%, and 0.76% of the total recording duration (14,400 s), respectively. In addition, the EEG device used in this study (PLUG) employs active electrodes with a short electrode–preamplifier distance, which reduces susceptibility to electromagnetic noise and contributes to stable signal acquisition.

**・Minimum usable data duration**

For data inclusion, a minimum of 2 minutes of artifact-free EEG data was required for each condition. Recordings not meeting this criterion were excluded from analysis. For each recording, 135 seconds of EEG data were initially obtained. From these, a 120-second segment was selected for analysis. When no major artifacts were present, a continuous 120-second segment was extracted. When artifacts were identified, contaminated portions were removed, and a total of 120 seconds of artifact-free data were retained for analysis.

**・Quantification of blinks and muscle artifacts**

Eye blinks and muscle artifacts were not quantitatively measured. However, due to electrode placement near Cz, blink-related artifacts were less prominent compared to frontal recordings. Residual artifacts were minimized through visual inspection and segment rejection.

**Figure S1：Power spectral density (PSD) of EEG.**

**A.** Representative power spectral density (PSD) from a single participant. The top panels are at Cz, middles are at C3 and bottoms are at C4. **B.** Group-level averaged power spectral density (PSD) across participants. Solid lines represent the group mean normalized power, and shaded areas indicate variability across participants (standard deviation). **A.B** The panels are arranged from left to right as Clock-Morning, Clock-Evening, Self-Moring and Self-Evening.

**Figure S2： The heatmaps of impedance data from Cz, C3 and C4.**

The top panels represent data for ECs and the bottom panels represent data for LCs. Color intensity represents impedance values (kΩ), with lighter colors indicating higher impedance. Impedance was continuously monitored during data acquisition, and segments with impedance exceeding 50 kΩ were excluded from subsequent analyses.

1. Mori F, Masaaki H, Seitaro I, Junichi U. Design and validation of a novel portable electrode holder for motor-related EEG measurement. bioRxiv 2026.03.02.705772;
2. David Hairston W, Whitaker KW, Ries AJ, Vettel JM, Cortney Bradford J, Kerick SE, McDowell K. Usability of four commercially-oriented EEG systems. J Neural Eng. 2014 Aug;11(4):046018.
3. Ferree, T. C., Luu, P., Russell, G. S., & Tucker, D. M. (2001). Scalp electrode impedance, infection risk, and EEG data quality. Clinical neurophysiology, 112(3), 536-544.
